# Dose-dependent effects of dietary quercetin supplementation on growth performance, nutrient digestibility, meat composition, bone mineralization, and profitability in broilers

**DOI:** 10.5455/javar.2026.m1007

**Published:** 2026-03-05

**Authors:** Md. Abu Saied, Syed Abu Yousuf, Md. Aliar Rahman, Khan Md. Shaiful Islam, Bodhi Agustono, Rakhi Chowdhury

**Affiliations:** 1 Department of Animal Nutrition, Bangladesh Agricultural University, Mymensingh, Bangladesh; 2 Veterinary Medicine, Faculty of Health, Medicine and Life Sciences, Universitas Airlangga, Indonesia

**Keywords:** Quercetin, broilers, performance, digestibility, bone mineralization, profit

## Abstract

**Objectives:** This study aimed to determine the optimal dose of quercetin (plant origin) on growth performance, nutrient digestibility, meat composition, shank mineralization, and production costs in broilers.

**Materials and Methods:** A total of 180 day-old, mixed-sex Ross 308 chicks were randomly assigned to four dietary groups, with five replicates of nine birds per group. The corn-soy-based basal diet provided 2998 kcal metabolizable energy (ME)/kg and 22.94% crude protein (CP) in the starter diet and 3120 kcal ME/kg and 21.25% CP in the grower diet. The basal diet was supplemented with quercetin at 0.00, 0.40, 0.80, or 1.20 gm/kg, corresponding to the 0.00Q (control), 0.40Q, 0.80Q, and 1.20Q groups, respectively.

**Results:** Quercetin supplementation at increasing levels improved feed intake and body weight gain, increased conversion ratios (feed, CP, and ME), and increased meat fat content, with the 1.20Q group showing the greatest effects (*p* ≤ 0.05). However, there were no significant differences among the quercetin-supplemented groups. Moreover, quercetin supplementation had no impact on broiler dressing yield (DY), drip loss, meat components (dry matter (DM), CP, and ash), shank DM, and ash percentages (*p* > 0.05). Shank dry weight, DM, ash yield, and nutrient digestibility were significantly improved in the 1.20Q group compared to the control and 0.40Q groups (*p* < 0.05). Quercetin supplementation at increasing levels significantly increased feed and quercetin costs but improved profit and the benefit-cost ratio (BCR), with the 1.20Q group showing the greatest improvement (*p* < 0.05).

**Conclusions:** Supplementation at 1.20 gm quercetin/kg diet improved growth performance, digestibility, shank mineralization, and BCR, but had no significant effect on DY and meat composition except for fat content.

## 1. Introduction

Flavonoids (bioflavonoids) are a diverse group of phytochemicals generally present in all plant species at variable levels. They exhibit a wide range of health-promoting properties, including antimicrobial, anti-inflammatory, antioxidant, and hepatoprotective effects [1]. Due to these advantageous properties, they are beneficial to human health and broiler production [2]. For example, flavonoid supplementation has been shown to improve broiler performance by increasing average daily gain, enhancing feed efficiency through a reduced feed conversion ratio (FCR), and reducing mortality rates, without exerting any adverse impacts on broiler, human, or environmental health. In addition, increasing research interest has focused on flavonoids due to their diverse biological activities, particularly their potential to mitigate the detrimental effects of oxidative stress, as well as their wide availability and cost-effectiveness [3].

Quercetin is a flavonoid compound that belongs to the class of flavonols. It can be found in most edible fruits, such as apples, and is mostly present in pomace, which is non-toxic, non-carcinogenic, and harmless to the body [4].

Additionally, it is added to broiler feed as a phytogenic additive due to its diverse potential benefits, including improved oxidation stability, meat quality, immune function, and anti-inflammatory properties [5]. As a potent free radical scavenger, it protects against organ damage and inhibits the effects of oxidative stress [6, 7]. Additionally, it possesses immunomodulatory and anti-inflammatory properties [8]. Feeding broiler chickens a diet containing quercetin dihydrate (0.00, 0.50, and 1.00 gm/kg diet) didn’t impact their body weight (BW) and feed intake (FI) but negatively affected FCR [5]. In contrast, dietary supplementation of commercial quercetin (purity: 97%) at different levels (0.00, 0.25, 0.50, or 1.00 gm/kg diet) has resulted in linear improvements in BW gain, enhanced intestinal morphology and serum antioxidant, and healthier gut health by enhancing *Lactobacillus* populations but has no impact on nutrient digestibility in broilers [9]. In contrast, reagent grade quercetin (Sigma-Aldrich) supplementation in the diet of broiler chickens at different levels (0.00, 0.20, 0.40, or 0.80 gm/kg diet) has no impact on BW gain, FCR, but linearly improves FI, gut beneficial microbiome, antioxidant related gene expression, and serum overall antioxidant status and reduces pathogenic gut microbiome [10].

Additionally, supplementing reagent-grade Sigma-Aldrich quercetin (purity: 97%) at a level of 0.00, 0.20, 0.40 or 0.60 gm/kg diet has no significant impact on growth [11], but 0.60 gm of the exact source of quercetin per kg of diet improves the bone mineralization in broilers via facilitating the gene expression linked to the Wnt signaling pathway [12]. Besides, quercetin can also enhance calcium absorption in the small intestine and increase vitamin D receptor activity in humans [13]. It is observed that quercetin’s efficacy is dose-dependent. The type of quercetin, like higher dietary inclusion levels of commercial quercetin (e.g., 0.50, 0.60, 0.80, or 1.00 gm/kg), has been shown to either improve gut health via modulating gut morphometry and microbiome, up-regulate genes associated with antioxidant defense, boost serum antioxidant status, or support bone mineralization. However, its influence on FI, BW gain, FCR, and nutrient digestibility remains inconsistent across different studies [5, 9, 10, 11]. Although these quercetins at similar supplementation levels have demonstrated beneficial effects on serum antioxidant status and the expression of genes associated with antioxidant defense, their influence on meat composition, especially meat fat content, has not yet been investigated. Furthermore, to our knowledge, no study has yet assessed the cost-effectiveness of supplementing broilers with plant-derived quercetin in the context of broiler production. Therefore, this study was designed to use a wider dose range of plant-originated quercetin (0.00, 0.40, 0.80, and 1.20 gm/kg diet) to determine the most effective supplementation level with significant impacts on growth performance, nutrient digestibility, meat composition, shank mineralization, and production costs in broilers.

## 2. Materials and Methods

### 2.1. Ethical clearance

The procedures of this research were approved by the Ethical Standards Committee at the Bangladesh Agricultural University (BAU) Research System (BAURES/ESRC/58/2024), Bangladesh.

### 2.2. Experimental birds, design, and diet

A total of 180 straight-run day-old broiler chicks (Ross 308) were procured from C.P. Bangladesh Co. Ltd. Using these chicks, a trial was conducted at the poultry rearing unit of BAU, belonging to the Department of Animal Nutrition. The chicks (46.24 ± 0.86 gm/bird) were randomly divided into 04 dietary groups (45 birds/group) with 5 replications (9 birds/replication). The control group received a corn–soy-based hand-mix mash diet without quercetin supplementation (0.00Q). The control diet contained metabolizable energy (ME) of 2,998 kcal/kg and crude protein (CP) of 22.94% during the starter phase and ME of 3,120 kcal/kg with CP of 21.25% during the grower phase (Ross 308 guideline). The remaining three groups received the same mash diet supplemented with quercetin (gm/kg) at levels of 0.40, 0.80, and 1.20 of the diet, designated 0.40Q, 0.80Q, and 1.20Q, respectively. The quercetin powder (derived from *Sophora japonica*) was 98% pure. The detailed nutrient composition of the basal mash diet is presented in Table 1.

**Table 1.** Ingredients and nutritional composition of basal diets (mash form).

Ingredients	Amounts (%)		Ingredients	Proximate composition (%)	
	Starter (1–14 days)	Grower (15–35 days)		Starter diet	Grower diet
Corn (crushed)	52.57	56.80	Crude protein	22.94	21.25
Protein concentrate*	5.29	7.00	Lysine^‡^	1.46	1.28
Rice polish	1.00	1.00	Methionine^‡^	0.58	0.67
Soybean meal	34.50	27.50	Crude fibre	4.12	4.02
Limestone	0.50	0.40	Ash	7.12	7.45
Di-calcium phosphate	1.71	1.60	Calcium^‡^	0.98	1.00
Soybean oil	3.00	4.00	Av. phosphorus^‡^	0.47	0.43
Lysine	0.24	0.25	ME^‡^ (kcal/kg)	2998	3120
Methionine	0.19	0.30			
Choline chloride	0.10	0.10			
Phytase	0.10	0.10			
Salmonella killer	0.10	0.10			
Toxin binder	0.10	0.10			
Broiler premix*	0.30	0.45			
Salt	0.30	0.30			

Av.: Available; ME: Metabolizable energy; *: Rahman et al. [14]; ‡: calculated value.

### 2.3. Management and data recording

All the cages were set up with feeders and drinkers, which were cleaned regularly and supplied with proposed diets and clean water, respectively. Birds had no restrictions on their mash diet and had access to fresh, clean drinking water. During the brooding period, the temperature was maintained at 33°C in the 1^st^ week and gradually reduced by 3°C per week over the subsequent two weeks. Thereafter, the birds were reared at a temperature range of 25°C to 27°C for the rest of the days. Throughout this period, electric bulbs were used as a source of artificial lighting. In addition, sawdust served as litter material, spread evenly to a depth of 2 cm, wilted regularly, and was replaced every week. The birds were vaccinated against Newcastle Disease on the 5^th^ day and Infectious Bursal Disease (Gumboro) on the 12^th^ day of the trial. During this trial, strict biosecurity measures were maintained.

The body weight (BW) of the birds was recorded upon arrival and subsequently every week. Daily feed intake (FI) and mortality were also recorded. At the end of the trial, total FI, CP intake, and ME intake were divided by BW gain to calculate the feed conversion ratio (FCR), crude protein conversion ratio (CPCR), and metabolizable energy conversion ratio (MECR), respectively [14].

### 2.4. Apparent nutrient digestibility, slaughtering, and samples analysis

Apparent nutrient digestibility was measured by adding 0.50% chromium oxide (Cr₂O₃) to the feed during the last seven days (day 28–35) of the trial. The first four days served as an adaptation period. During the final three days of the experiment, excreta samples were collected daily, oven-dried at 60–65°C, and subsequently stored at –4°C until further analysis. Additionally, the daily dry matter (DM) of fresh feces was determined according to the AOAC [15]. Afterwards, the birds were slaughtered at 35 days by halal methods (cervical dislocation) and waited until complete bleeding. Then, the birds were processed, and dressing yield (DY) was calculated, excluding skin, visceral organs, head, feet, and giblets (heart, liver, neck, and gizzard), as described by Siddik et al. [16]. Then, drip loss was determined by recording the weight of breast muscle samples before and after 24 h of chilled storage at 4°C, and the difference in weight was calculated and expressed as a percentage of the initial weight.

Fresh breast meat was minced, and dried feces and mash diet (starter and grower) were ground through a 1.0 mm screen by a grinder. These samples were analyzed for the determination of DM, CP, and ether extract (EE), and for the calculation of nitrogen-free extract (NFE) in accordance with AOAC [15]. In addition, chromium oxide (Cr₂O₃) of feed and feces samples was detected according to Akter et al. [17]. Then, apparent nutrient digestibility was calculated using the following equation, considering all nutrient percentages on a DM basis:


\[
{\rm Apparent\ digestibility\ (\%)} = 100-\left({\frac{{{{\rm N}_{\rm f}} \times {{\rm M}_{\rm d}}}}{{{{\rm N}_{\rm d}} \times {{\rm M}_{\rm f}}}}\; \times \;100} \right)
\]

Where, N_f_ = nutrient percentage in the feces, N_d_ = nutrient percentage in the diet, M_d_ = marker (chromium) percentage in the diet, M_f_ = marker (chromium) percentage in the feces.

Following the removal of muscles and tendons using 70°C hot water, the shank samples were dried at 105°C for 24 h, weighed, and subsequently ashed at 600°C for an additional 24 h [15]. After recording the shank dry weight and determining the DM and ash percentages of the shank, the shank DM and ash yields were calculated by multiplying the dry weight of the shank by their respective DM and ash percentages.

### 2.5. Economic analysis

Cost analysis and benefit–cost ratio (BCR) were conducted by accounting for the costs of feed ingredients, test substances (quercetin), chicks, and other expenses, including vaccination, litter, labor, and electrical equipment, as well as the selling price of live birds, following the procedure described by Chowdhury et al. [18].

### 2.6. Statistical analysis

Initially, the raw data were organized in Excel and then analyzed using SPSS (version 22) for a one-way analysis of variance. The significance of the difference among means was determined using Tukey’s HSD test (1953), and differences at *p* ≤ 0.05 were considered statistically significant. Moreover, data were analyzed using an orthogonal polynomial contrast to determine the optimal quercetin dose.

## 3. Results

### 3.1. Growth performance traits

Dietary supplementation of quercetin at different doses (0.00, 0.40, 0.80, and 1.20 gm/kg diet) resulted in a linear improvement in growth performance traits, including final BW, BW gain, and FI, accompanied by better FCR, CPCR, and MECR (Table 2). The most pronounced effects were observed in birds receiving 1.20 gm quercetin per kg diet (1.20Q group; *p* < 0.05), which exhibited approximately 5% higher FI and 9% greater BW and BW gain compared with the control group (0.00 gm quercetin/kg diet). Birds supplemented with 0.40 or 0.80 gm of quercetin per kg of diet exhibited improved growth performance relative to the control, although the difference was not statistically significant (*p* > 0.05).

**Table 2.** Effects of different doses of quercetin supplementation on growth performance of broilers.

Variables^2^	Quercetin (gm/kg basal diet^1^)				*p*-value^3^			
	0.00 (Control)	0.40	0.80	1.20	Combined	L	Q	C
Final BW (gm/bird)	1256^b^ ± 33.2	1283^ab^ ± 50.3	1324^ab^ ± 38.9	1369^a^ ± 46.5	0.05	0.01	0.71	0.91
BW gain (gm/bird)	1216^b^ ± 26.9	1236^ab^ ± 50.3	1278^ab^ ± 38.9	1323^a^ ± 46.5	0.05	0.01	0.62	0.87
Feed intake (gm/bird)	2221 ± 59.67	2240 ± 50.12	2281 ± 54.39	2321 ± 63.7	0.22	0.05	0.76	0.88
FCR	1.82^a^ ± 0.01	1.81^ab^ ± 0.04	1.78^ab^ ± 0.01	1.75^b^ ± 0.01	0.04	0.01	0.63	0.85
CPCR	0.402^a^ ± 0.004	0.399^ab^ ± 0.009	0.392^ab^ ± 0.003	0.386^b^ ± 0.004	0.04	0.01	0.62	0.85
MECR	5.50^a^ ± 0.05	5.46^ab^ ± 0.12	5.37^ab^ ± 0.04	5.29^b^ ± 0.05	0.04	0.01	0.62	0.85

^1^Corn-soybean meal-based hand-mixed basal diet containing 2998 kcal ME/kg feed and 22.94% CP in the starter phase and 3120 kcal ME/kg feed and 21.25% CP in the grower phase. ^2^BW: Body weight, ADG: Average daily gain, FCR: Feed conversion ratio, CPCR: Crude protein conversion ratio, MECR: Metabolizable energy conversion ratio, gm: gram. ^3^L: Linear, Q: Quadratic, C: Cubic, ^ab^ Means values with dissimilar superscripts differ significantly (*p* ≤ 0.05).

### 3.2. Apparent nutrient digestibility

Quercetin supplementation in the basal diet at levels of 0.00, 0.04, 0.08, and 1.20 gm/kg diet presented a significant linear increase (*p* < 0.05) in the apparent digestibility of DM, CP, EE, and NFE (Table 3). Birds receiving 1.20 gm quercetin/kg diet exhibited approximately 4%, 5%, 6%, and 4% higher digestibility of DM, CP, EE, and NFE, respectively, compared with the birds fed the control diet. Supplementation with 0.40 and 0.80 gm quercetin per kg diet also resulted in significant improvements in DM and EE digestibility (*p* < 0.05). In contrast, no significant changes in CP and NFE digestibility were observed relative to the control (*p* > 0.05).

**Table 3.** Effects of different doses of quercetin supplementation on apparent nutrient digestibility of broilers.

Variables (Percentage)	Quercetin (gm/kg basal diet^1^)				*p* -value^2^			
	0.00 (Control)	0.40	0.80	1.20	Combined	L	Q	C
Dry matter	89.04^c^ ± 0.06	90.85^b^ ± 0.70	91.84^ab^ ± 0.22	92.81^a^ ± 0.63	< 0.01	< 0.01	0.17	0.53
Crude protein	65.38^b^ ± 0.48	66.52^b^ ± 0.93	66.62^b^ ± 1.13	68.90^a^ ± 0.32	0.004	0.001	0.24	0.15
Ether extract	85.09^c^ ± 0.46	87.73^b^ ± 0.36	88.08^b^ ± 0.78	90.06^a^ ± 0.62	< 0.01	< 0.01	0.36	0.03
Nitrogen-free extract	84.13^b^ ± 0.74	85.23^b^ ± 0.70	85.65^b^ ± 0.51	87.64^a^ ± 0.61	< 0.01	< 0.01	0.002	0.02

^1^Corn-soybean meal-based hand-mixed basal diet containing 2998 kcal ME/kg feed and 22.94% CP in the starter phase and 3120 kcal ME/kg feed and 21.25% CP in the grower phase. ^2^L: Linear, Q: Quadratic, C: Cubic, ^a-c^ Means values with dissimilar superscripts differ significantly (*p* ≤ 0.05).

### 3.3. Dressed yield and meat composition

Dietary supplementation of quercetin at different doses had no significant effect on DY, drip loss, or breast meat proximate compositions, including DM, CP, and ash (*p* > 0.05; Table 4), except for EE (*p* < 0.05). Increasing quercetin inclusion resulted in a progressive reduction in meat EE (fat) content, with the most pronounced reduction observed in birds receiving 1.20 gm quercetin/kg diet (1.20Q group; *p* < 0.05), which was approximately 10% compared with the birds fed the control diet (0.00Q group).

**Table 4.** Effects of different doses of quercetin supplementation on broilers’ carcass traits and breast meat composition.

Variables^2^ (%)	Quercetin (gm/kg basal diet^1^)				*p* -value^3^			
	0.00 (Control)	0.40	0.80	1.20	Combined	L	Q	C
DY	58.51 ± 1.60	59.62 ± 1.84	59.33 ± 1.37	59.69 ± 0.83	0.74	0.41	0.66	0.60
Drip loss	2.58 ± 0.18	2.50 ± 0.07	2.48 ± 0.11	2.44 ± 0.02	0.54	0.17	0.77	0.85
Dry matter	25.43 ± 0.37	25.73 ± 0.60	25.13 ± 0.81	24.88 ± 0.96	0.53	0.26	0.51	0.52
Crude protein	24.99 ± 0.74	25.11 ± 0.88	25.30 ± 0.64	25.94 ± 0.78	0.48	0.16	0.57	0.85
Ether extract	1.07^a^ ± 0.02	1.06^ab^ ± 0.01	1.01^ab^ ± 0.02	0.96^b^ ± 0.03	0.03	0.01	0.53	0.87
Ash	1.47 ± 0.18	1.48 ± 0.17	1.46 ± 0.13	1.48 ± 0.11	0.99	0.96	0.96	0.86

^1^A corn-soybean meal-based hand-mixed diet containing 2998 kcal ME/kg feed and 22.94% CP in the starter phase and 3120 kcal ME/kg feed and 21.25% CP in the grower phase. ^2^DY: Dressed yield without skin, visceral organs, head, feet, and giblets; %: Percentage; gm: gram. ^3^L: Linear, Q: Quadratic, C: Cubic, ^ab^ Means values with dissimilar superscripts differ significantly (*p* ≤ 0.05).

### 3.4. Bone mineralization

Dietary supplementation of quercetin at different doses resulted in a significant linear improvement (*p* < 0.05) in shank dry weight, DM, and ash yield, whereas no noteworthy effects were observed on shank DM and ash percentages (*p* > 0.05; Table 5). Birds receiving 1.20 gm quercetin per kg diet exhibited approximately 15%, 16%, and 20% higher shank dry weight, DM, and ash yield, respectively, compared with those fed the control diet. However, no significant differences were observed between the 1.20Q and 0.80Q groups, and similarly, the 0.80Q, 0.40Q, and control groups did not differ significantly in these shank-related parameters.

**Table 5.** Effects of different doses of quercetin supplementation on shank mineralization of broilers.

Variables^2^	Quercetin (gm/kg basal diet^1^)				*p*-value^3^			
	0.00 (Control)	0.40	0.80	1.20	Combined	L	Q	C
Dry weight (gm)	1.75^b^ ± 0.10	1.76^b^ ± 0.10	1.88^ab^ ± 0.08	2.02^a^ ± 0.05	0.01	0.003	0.26	0.59
Dry matter (%)	97.97 ± 0.29	98.25 ± 0.79	98.07 ± 0.52	98.19 ± 0.22	0.91	0.72	0.79	0.57
Dry matter yield (gm)	1.71^b^ ± .09	1.72^b^ ± .09	1.85^ab^ ± 0.07	1.98^a^ ± 0.04	0.01	0.002	0.23	0.61
Ash (%)	35.78 ± 1.87	35.54 ± 1.39	35.81 ± 0.99	36.92 ± 0.99	0.63	0.32	0.42	0.93
Ash yield (gm)	0.61^b^ ± 0.06	0.61^b^ ± 0.05	0.66^ab^ ± 0.09	0.73^a^ ± 0.01	0.03	0.006	0.20	0.80

^1^Corn-soybean meal-based hand-mixed basal diet containing 2998 kcal ME/kg feed and 22.94% CP in the starter phase and 3120 kcal ME/kg feed and 21.25% CP in the grower phase. ^2^ gm: gram, %: Percentage. ^3^L: Linear, Q: Quadratic, C: Cubic, ^ab^ Means values with dissimilar superscripts differ significantly (*p* ≤ 0.05).

### 3.5. Cost-benefit analysis

**Figure 1.**
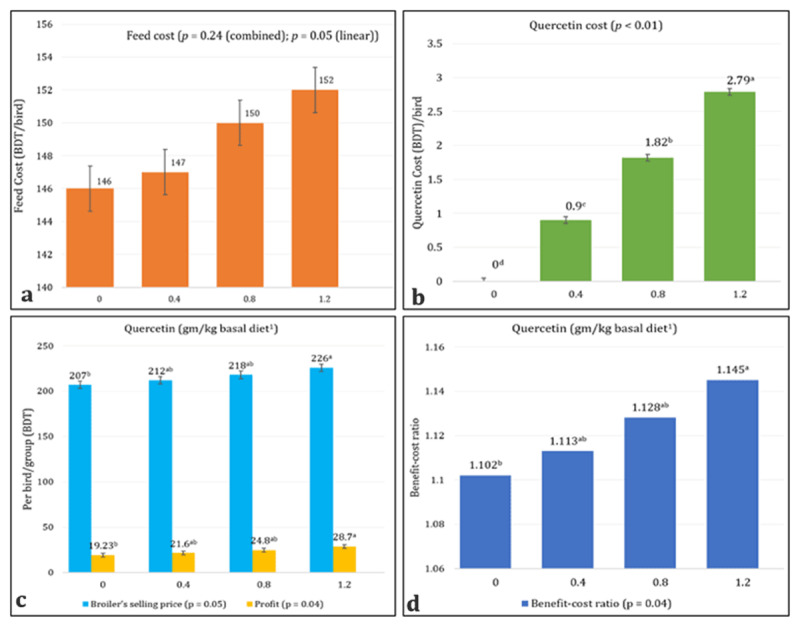
Effects of different doses of quercetin supplementation on (a) feed cost, (b) quercetin cost, (c) selling price and profit, and (d) benefit-cost ratio of broilers (in BDT). ^1^Corn-soybean meal-based hand-mixed basal diet containing 2998 kcal ME/kg feed and 22.94% CP in the starter phase and 3120 kcal ME/kg feed and 21.25% CP in the grower phase. ^a–d^ Means values with dissimilar superscripts differ significantly (*p* ≤ 0.05).

Dietary supplementation of quercetin at incremental doses (0.00, 0.40, 0.80, and 1.20 gm/kg of the basal diet) resulted in a linear improvement in economic performance parameters including feed cost, quercetin cost, broiler selling price, profit, and BCR, with the 1.20Q group showing significant differences compared to the 0.00Q (control) group (*p* < 0.05; Figure 1). Although feed cost (Figure 1a) showed a linear trend (*p* = 0.05), the combined effect was not statistically significant (*p* = 0.24). In contrast, quercetin cost (Figure 1b) differed significantly among the groups, with the highest and lowest values recorded in the 1.20Q and control (0.00Q) groups, respectively. The 1.20Q group also showed significantly higher broiler selling price, profit (Figure 1c), and BCR (Figure 1d) relative to the control group. No significant differences were observed in these economic parameters among the control (0.00Q), 0.40Q, and 0.80Q groups, or among the 0.40Q, 0.80Q, and 1.20Q groups (*p* > 0.05).

## 4. Discussion

Final BW and BW gain linearly increased with quercetin supplementation in the doses of 0.00, 0.40, 0.80, and 1.20 gm per kg diet, with a considerable performance in birds of the 1.20Q group. Moreover, the birds fed quercetin showed higher linear FI and apparent nutrient digestibility, and better FCR, CPCR, and MECR responses; significant effects were observed in the 1.20Q group, which could partially explain the previous findings [9]. These partial similarities may be due to differences in quercetin dose, purity, and origin [10, 11, 19, 20]. Furthermore, inclusion of quercetin (gm/kg diet) at 0.00, 0.20, 0.40, and 0.80 [10] and 0.20, 0.40, and 0.60 [11] in birds did not affect BW gain and FCR, which is contrary to the present experiment. This difference may be due to the source of origin and the lower, narrower range of quercetin supplementation compared to the present study [11, 20]. In the current study, plant-derived (*S. japonica*) 98% quercetin was given over a broader dose range, for example, 0.40 gm per kg and higher doses of 0.00–1.20 gm per kg, which may be responsible for the attainment of statistically significant values in the 1.20Q group as found in other previous work [20]. In the present study, a dose-dependent increase in FI with increasing levels of quercetin was observed, which may be attributed to its antioxidant, hepatoprotective, and immunostimulatory properties, potentially facilitating the secretion of digestive enzymes [9].

Broilers fed micellar quercetin at the rates of 0.00, 0.25, 0.50, and 1.00 gm per kg diet derived from *S. japonica* did not show a significant effect on DM, nitrogen, and energy digestibility [20]. In contrast, the current study contradicts this. This inconsistency could be due to several factors, including differences in dose and quercetin formulation, which may have contributed to quercetin’s inability to have a beneficial impact on gut microbiota and nutrient utilization [20]. In addition, like our present study results, birds fed 0.60 gm quercetin per kg diet showed better digestibility for DM and energy [21, 22]. This finding also partly aligns with the current study’s results, which may be attributed to a higher number of beneficial intestinal bacteria that enhanced nutrient utilization [23]. In addition, birds fed with quercetin (gm/kg diet) at different levels (0.00, 0.25, 0.50, and 1.00 or 0.00, 0.20, 0.40, and 0.80) have shown an increase in the *Lactobacillus* population in a linear response [9, 10] and increased villus height and crypt depth in the jejunum [10], which may help in lowering the risk of dysbiosis and improving nutrient utilization [17]. Furthermore, quercetin has strong antioxidant capacity, which stimulates the secretion of digestive enzymes, enhances superoxide dismutase activity, mitigates oxidative stress, reduces gut inflammation, and promotes a favorable gut microbiome [24].

These beneficial effects contribute to improved intestinal integrity, protection of nutrients from oxidative damage, and strengthened immune responses, thereby facilitating the establishment of beneficial microbial communities and ultimately enhancing nutrient digestibility and absorption [10, 24]. These favorable changes resulted in improved FCR, CPCR, MECR, and BW gain in our current study. However, it was found that the increase in final BW and BW gain depended on the quercetin dose; that is, with higher doses, these effects improved.

The meat fat content decreased with quercetin up to a linear level, which was more pronounced at a dose of 1.20 gm quercetin per kg diet. This observation is also in agreement with the report that the supplementation of 0.60 gm per kg diet with reagent-grade quercetin (Sigma-Aldrich) significantly decreased the fat content in the breast muscle of broilers [25]. Furthermore, administration of a high level of quercetin supplementation in feed resulted in a significant reduction in intraperitoneal fat content, possibly because quercetin affects lipid metabolism, thereby reducing the ability of fat to deposit in broiler meat [9]. It attenuates triacylglycerol accumulation in broiler liver cells by upregulating the PPARα signaling pathway, which controls lipid decomposition, and thereby decreasing lipid accumulation [26]. In addition, quercetin inhibited adipogenesis by increasing the expression of adipose triglyceride lipase and hormone-sensitive lipase and decreasing fatty acid synthase, lipoprotein lipase, and adipocyte fatty acid-binding protein in OP9 mouse stromal cells [27]. These studies suggest that higher levels of quercetin supplementation may increase lean meat by reducing its fat content. The fatty acid profile of the meat was not evaluated in this study; therefore, further research is recommended to investigate meat quality, especially fatty acid composition.

The bone DM% and ash% were not affected by quercetin supplementation; however, quercetin significantly enhanced the dry weight, DM and ash yield of the shank, with a more remarkable effect in the 1.20Q group. These results agree with the results of the study [12], which indicated that reagent-grade quercetin (Sigma-Aldrich) supplementation at 0.60 gm per kg significantly increased the relative and unit weight of phosphorus content and bone mineral density (BMD) of the tibia in broilers. In addition, daily administration of quercetin at 0.10 gm per kg [28] or 25 gm per kg BW [29] increased BMD, and the weight index of rats and BMD in ovariectomized rats, respectively. These benefits are likely due to supplementation with quercetin in broilers, which downregulated *Wnt-5a, CAMK2B, CAMK2D, CAMK2G, PLCB4*, and *NFATC1*, but upregulated *PRKCA*, thereby promoting tibia development and regulating calcium-phosphorus metabolism [12]. Additionally, quercetin inhibited osteoclast differentiation by suppressing the nuclear factor κB (NF-κB) and activator protein 1 (AP-1) signaling pathways [30]. Collectively, the positive changes in the bone (e.g., shank dry weight, DM, and ash yield) found in quercetin-supplemented birds from this study might be linked to some alterations in NF-κB, AP-1, and Wnt gene signaling pathways as well as bone mineralization.

There was a linear increase in profit and BCR in quercetin-supplemented birds, as depicted by the 1.20Q group, which differed from a flavonoid blend given at various levels [17]. This difference may be due to several factors, such as the higher cost of the flavonoid blend used and the relatively lower BW gain in the previous trial [17]. Interestingly, birds fed lower levels of herbal supplementation showed higher profits [31], supporting the present results. The 1.20Q group displayed the highest profit and BCR among all groups in this study, which might be attributed to increased nutrient digestibility and better feed, protein, and energy conversion ratios, as well as markedly higher BW gain and a higher broiler selling price. The price of quercetin was considerably higher in this group, but this difference did not affect the overall feed cost.

## 5. Conclusions

Birds receiving quercetin at increasing levels (0.00, 0.40, 0.80, or 1.20 gm/kg basal diet) resulted in linear improvements in feed intake, body weight gain, and feed conversion ratio, with considerable effects observed in birds supplemented with 1.20 gm quercetin/kg diet. Quercetin supplementation had no significant impact on dressing yield, shank dry matter and ash percentages, or meat nutrient content, except for a reduction in meat fat content, which was substantial in birds fed 1.20 gm of quercetin per kg of diet. Furthermore, birds receiving 1.20 gm quercetin per kg diet exhibited considerably improved shank dry weight, dry matter, ash yield, and nutrient digestibility. Although quercetin supplementation increased feed and quercetin costs, it led to improved profitability and a better benefit-cost ratio, with a substantial improvement observed in birds receiving 1.20 gm quercetin per kg of diet.

## Data Availability

The data presented in this study are available from the corresponding author upon reasonable request.

## List of abbreviations

AOAC, association of official analytical chemists; AP-1, activator protein-1; BCR, benefit–cost ratio; BMD, bone mineral density; BW, body weight; CAMK2B, calmodulin-dependent protein kinase II beta; CAMK2D, calmodulin-dependent protein kinase II delta; CAMK2G, calmodulin-dependent protein kinase II gamma; CP, crude protein; CPCR, crude protein conversion ratio; FCR, feed conversion ratio; DM, dry matter; DY, dressing yield; FI, feed intake; EE, ether extract; ESRC, ethical standards of the research committee; gm, gram; h, hours; kg, kilogram; ME, metabolizable energy; MECR, metabolizable energy conversion ratio; NFE nitrogen-free extract; NFATC1, nuclear factor of activated T-cells; NF-κB, nuclear factor kappa-light-chain-enhancer of activated-B cells; PLCB4, phospholipase-C beta 4; PPARα, peroxisome proliferator–activated receptor alpha; PRKCA, protein kinase-C alpha; Wnt, wingless/integrated.
